# A multilayered electrospun graft as vascular access for hemodialysis

**DOI:** 10.1371/journal.pone.0185916

**Published:** 2017-10-12

**Authors:** D. Radakovic, J. Reboredo, M. Helm, T. Weigel, S. Schürlein, E. Kupczyk, R. G. Leyh, H. Walles, J. Hansmann

**Affiliations:** 1 Department of Thoracic and Cardiovascular Surgery, University Hospital Wuerzburg, Josef-Schneider-Straße 2, Wuerzburg, Germany; 2 Department of Tissue Engineering and Regenerative Medicine, University Hospital Wuerzburg, Roentgenring 11, Wuerzburg, Germany; 3 Fraunhofer Institute for Silicate Research, Neunerplatz 2, Wuerzburg, Germany; Politecnico di Milano, ITALY

## Abstract

Despite medical achievements, the number of patients with end-stage kidney disease keeps steadily raising, thereby entailing a high number of surgical and interventional procedures to establish and maintain arteriovenous vascular access for hemodialysis. Due to vascular disease, aneurysms or infection, the preferred access—an autogenous arteriovenous fistula—is not always available and appropriate. Moreover, when replacing small diameter blood vessels, synthetic vascular grafts possess well-known disadvantages. A continuous multilayered gradient electrospinning was used to produce vascular grafts made of collagen type I nanofibers on luminal and adventitial graft side, and poly-ɛ-caprolactone as medial layer. Therefore, a custom-made electrospinner with robust environmental control was developed. The morphology of electrospun grafts was characterized by scanning electron microscopy and measurement of mechanical properties. Human microvascular endothelial cells were cultured in the graft under static culture conditions and compared to cultures obtained from dynamic continuous flow bioreactors. Immunofluorescent analysis showed that endothelial cells form a continuous luminal layer and functional characteristics were confirmed by uptake of acetylated low-density-lipoprotein. Incorporation of vancomycin and gentamicin to the medial graft layer allowed antimicrobial inhibition without exhibiting an adverse impact on cell viability. Most striking a physiological hemocompatibility was achieved for the multilayered grafts.

## Introduction

Due to a growing number of patients with end-stage kidney disease, the number of surgical and interventional procedures required to establish and maintain arteriovenous vascular access to hemodialysis (HD) keeps rising. Currently, it is expected that the number of patients on HD is growing by 4 to 8% yearly worldwide, and diabetes mellitus (DM) is considered as one of the major causes thereof. [1] For patients suffering from DM, an autogenous arteriovenous (AV) vascular access is preferred. [2] However, many patients lack suitable vein grafts for this procedure and rely on chronic venous catheters or prosthetic AV grafts, which carry a lifetime risk of thrombosis and infection. [1] For these patients, autologous or allogenic transplantation is employed. Unfortunately, this technique is not always available and appropriate. To overcome the drawbacks entailed by the transplantation of native vessels, synthetic vascular grafts made from expanded polytetrafluoroethylene (ePTFE) or polyethylene terephthalate (Dacron^®^) are successfully applied to replace large diameter blood vessels (≥ 6 mm), however these grafts fail when used for the replacement of small diameter blood vessels. [3, 4] The prosthetic grafts show a mismatch in compliance compared to natural vessels, and the prosthetic materials are susceptible to infection for the graft’s lifetime. Moreover, intimal hyperplasia and thrombosis have been associated with inadequate endothelial cell coverage of the luminal surface of the vascular graft. [5] Thus, infection, intimal hyperplasia and thrombosis, for which patients on dialysis have a higher risk, often lead to graft failure and contribute to high costs of renal replacement therapy. [6, 7]

Tissue engineering represents an option to generate novel, matured grafts with properties comparable to native blood vessels. The concept of tissue-engineered vascular grafts (TEVG) is based on combining tubular scaffolds, autologous vascular cells, and suitable culture conditions that allow to mature the seeded scaffold to in-vivo-like blood vessels. As a major cause of graft failure are bacterial contaminations [8], TEVGs should provide an environment incompatible for bacteria to reduce the risk of infection after graft implantation. Otherwise, bacterial cells overtake and win the survival competition with the host cells, and a biofilm is formed, in which bacterial cells are protected against antimicrobial agents and the immune system. [9, 10] Moreover, the antibacterial mechanism that protects the graft should facilitate a low cytotoxicity for the seeded and the host cells.

For scaffold generation, welding techniques in combination with heat activation have been described to produce tubular scaffolds [11]. However, regarding structural design principle, such vascular scaffolds have only limited similarities compared to blood vessels. Furthermore, an orthogonal orientation of the scaffold components results in low axial elongation with lower strain at maximal stress and lower elastic modulus compared to native arteries. [12] An effective technique to produce vascular scaffolds is electrospinning. This technology allows the fabrication of continuous nano- and microscale filaments from natural and synthetic polymers, thereby increasing the possibility to match both, the biological and mechanical properties of an artery. From a structural perspective, electrospun scaffolds furthermore mimic the multilayered architecture of arterial walls. [13] Most striking, electrospun vascular grafts ensure a sufficient initial mechanical strength and stiffness, an appropriate structural integrity during tissue growth and remodeling, a microarchitecture suitable for cell attachment and subsequent cell migration into the matrix, and a controlled degradation and resorption kinetics, which is a prerequisite for tissue development. [14]

The aim of this study was to manufacture multilayered, electrospun vascular scaffolds that exhibit mechanical properties similar to native blood vessels. Therefore, a bidirectional electrospinning device with controlled process conditions was employed. In contrast to the tendency of developing non-cell adhesive vascular grafts using supramolecular polymers [15], we hypothesized that a sufficient endothelial lining can be achieved in vitro prior to vascular graft implantation, thereby facilitating a physiological blood-tissue interface. The electrospun vascular scaffolds were seeded with human microvascular endothelial cells (hmvECs) and matured under shear stress in a dynamic bioreactor system in order to achieve a sufficient endothelial lining. Furthermore, the polymers used for electrospinning were blended with antibiotics, enabling antimicrobial graft properties that reduce the risk of on-site infections.

## Materials and methods

### Fabrication of PCL and PCL/Collagen vascular grafts

**Fig 1:**
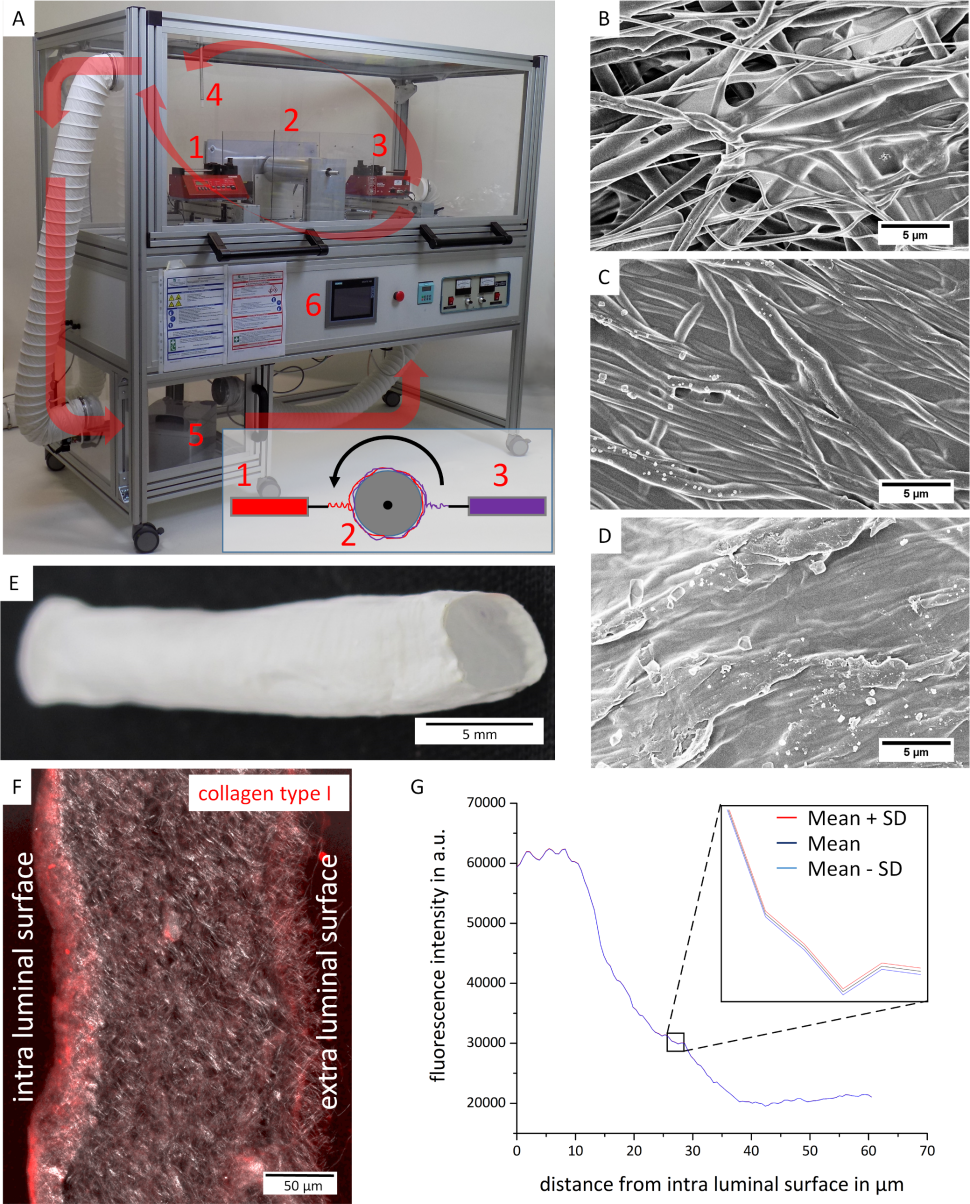
Generation of multilayered electrospun vascular scaffolds. (A) Bidirectional electrospinning in a custom-made closed electrospinning system. The inset displays the principle of the bidirectional electrospinning with two pumps harboring different polymer solutions. The arrows indicate the airflow that was controlled by ventilators. 1: syringe pump 1, 2: collector mandrel, 3: syringe pump 2, 4: humidity and temperature sensor, 5: humidifier, 6: user interface. (B-D) SEM images of scaffolds produced under different humidity levels (B = 30%, C = 50%, and D = 80%). (E) Due to the used collector mandrel, the vascular scaffolds had an inner diameter of 6 mm. (F) The multilayered architecture of the scaffold was shown by immunofluorescence staining of collagen type I. (G) A color image analysis confirmed a decaying collagen type I concentration inside the scaffold wall. Thereby, the concentration decreased from the luminal surface of the graft to the center of the scaffold wall. The inset in depicts the mean fluorescence intensity ± standard deviation (SD) in a higher magnification of the indicated region (n = 3).

Vascular grafts were fabricated by electrospinning of PCL and collagen type 1, which were separately dissolved. PCL was dissolved in 1,1,1,3,3,3-Hexafluoro-2-propanol (HFIP) at the concentration of 10%, 15% and 20% (w/v) and collagen type I acquired from rat skin in trifluoroethanol (TFE) at the concentration of 5% (w/v). A modified bidirectional gradient electrospinning process (Fig 1A) was used to fabricate multilayered vascular grafts with a symmetrical gradient structure, meaning that the concentration of two different polymers in a specific layer was controlled. The electrospinning setting included two syringe pumps, a high-voltage supply, and a rotating mandrel. A positive voltage of 8 kV was applied to the polymer solutions by the power supply (Gama High Voltage Research, Ormond Beach, FL, USA). Two syringe pumps (AL-1000, World Precision Instruments, Sarasota, FL, USA) were used in opposite directions. A stainless-steel mandrel (6 mm in diameter) with circumferential speed ranging from 0.162–0.315 m/s was used to collect vascular grafts. The distance between each syringe tip and the mandrel was 10 cm. To fabricate multilayered vascular grafts with gradient symmetrical structure, continuous bidirectional electrospinning was applied with flow rates (mL/h) for both polymer solutions as shown in Table 1. The junction between the collagen-type-I-blended layers and the pure medial PCL layer was generated by introducing a transitional zone composed of both PCL and collagen.

**Table 1:** Polymer flow rates during the electrospinning process of multilayered vascular grafts.

Step	PCL flow (mL/h)	Collagen type I flow (mL/h)	Duration (min)
I	0	0.4	30
II	0.2	0.4	15
III	0.4	0.2	15
IV	0.6	0	60
V	0.4	0.2	15
VI	0.2	0.4	15
VII	0	0.4	30

Therefore, both polymers were spun simultaneously and the volume flow of each polymer solution was linearly increased or decreased. This layer established a seamless interface in order to prevent delamination. During the electrospinning process, the temperature in electrospinning chamber was 22°C and relative humidity levels between 30% and 80% were tested. Following, the electrospun vascular grafts containing collagen type I were cross-linked using 10% hexamethylen-diisocyanat (HMDI, Sigma-Aldrich, Seelze, Germany) for 1 h. After that, scaffolds were washed 3x with 100% isopropanol for 20 min, and rinsed 3x in 70% ethanol in a sterile bench. As the last step, scaffolds were washed 3x in phosphate-buffer saline (PBS^-^) solution for 10 min. All scaffolds were sterilized using Gamma radiation before being seeded with hmvECs.

### Characterization of vascular grafts and mechanical properties

To characterize the morphology, size and orientation of the fibers of electrospun matrices, scanning electron microscopy (SEM, Supra 25, Zeiss, Oberkochen, Germany) was performed. The coating procedure was conducted using platinum. Images were acquired using a conventional SEM operating at accelerating voltage of 5 kV and working distance of 4 to 10 mm. The images were analyzed using ImageJ (open source Java image processing program) to determine the size of the electrospun fibers and pores.

Maximal longitudinal stress and strain were measured by clamping samples prepared in the same size (50 mm x 5 mm, n = 6). The thickness of the samples was estimated to 11 μm. A universal materials testing machine (Z010, Zwick, Ulm, Germany) was used at ambient temperature and humidity. A cross-head speed of 10 mm/min was selected for all the specimens tested until failure. The test was aligned to the American Society for Testing and Materials (ASTM) guideline D 882–02 (Standard Test Method for Tensile Properties of Thin Plastic Sheeting).

For burst pressure measurement, a 3-cm-long and 5-mm-thick glass tube was connected to a pressure recording device (Chair Tissue Engineering and Regenerative Medicine, Wuerzburg, Germany) on one side. The opposite side was hermetically sealed it with an electrospun vascular scaffold using suture threads. A manual filling with water was applied and the filling pressure was recorded until the scaffold wall failed. The maximum recorded pressure was set as burst pressure of the graft. All measurements were repeated on 4 samples for each type of vascular scaffold.

Suture retention strength was measured with tensile testing machine with a 200-N-load cell. To determine the suture retention strength, the vascular graft was cut normal to the long axis into small strips dimension of 20 mm x 5 mm. On one end, electrospun vascular scaffolds were clamped and on the other 5–0 prolene suture (Ethicon, USA) was passed through it and knotted 2 mm from the edge. The suture was pulled at a rate of 50 mm/min. The force required to pull the suture through the graft wall was recorded as suture retention strength. This test was repeated on 3 samples for each type of vascular scaffold.

Surface wettability of different vascular grafts was tested by measuring angle of water droplets by using contact angle measuring instrument (Contact angle system OCA, Dataphysics, Germany). Two different positions on 6 samples of each vascular graft were tested, and 0.02 mL deionized water was used for each measurement.

### Isolation and culture of human microvascular endothelial cells (hmvECs)

Human microvascular endothelial cells (hmvEC) were isolated from fresh biopsies of human prepuces. The procedure was approved by the ethical commission of the University of Wuerzburg (vote 182/10). Informed and written consent from either the patient or from the next of kin, caretakers, or guardians was obtained if the patient was underage. Briefly, skin biopsies (2 cm^2^ surface) was washed with PBS^-^ containing 1% antibiotics penicillin and streptomycin (pen/strep) and cut longitudinally. Following, the subcutis was removed with a sterile scalpel and washed again with PBS^-^. The tissue was then placed in dispase solution (4 U/mL) and incubated overnight. The next day, the epidermis and dermis were separated, dermis was washed with the solution of 0.1 vol-% Ethylenediaminetetraacetate in PBS^-^ (PBS^-^ /EDTA), and covered with 10 ml of a 0.05 vol-% trypsin in PBS^-^/EDTA solution (trypsin/EDTA). The dermis was incubated for 40 min in the incubator and trypsin activity was stopped by adding 25 vol-% fetal calf serum (FCS). Subsequently, the stripes were placed into a fresh petri dish filled with VascuLife^®^ (CellSystems GmbH, Troisdorf, Germany) and were crossed out ten times with gentle pressure on each side using a sterile scalpel blade. In order to free the cell suspension from smaller tissue pieces, the suspension was filtered through a cell strainer with 100 μm pore size into a 50 ml Falcon tube, then the suspension was centrifuged for 5 min at 1200 rpm and seeded in cell culture flasks. HmvECs were grown for 2 to 3 weeks of culture, and medium was exchanged every 3 days. In order to achieve highly pure endothelial cell culture, the cells were sorted using magnetic activated cell sorting—MACS of hmvEC (MACS CD31 microBead Kit, Miltenyi Biotec, Germany). After treatment of the cultured cells with trypsin, CD31^+^ hmvEC are immunolabeled with CD31 MicroBeads, before being loaded onto a column placed in the magnetic field of a MACS Separator. The magnetically labeled CD31^+^ cells were retained within the column. After removing the column from the magnetic field, the magnetically-retained CD31^+^ cells could be eluted as the positively selected cell fraction and directly taken into further hmvEC pure cell culture.

### Static culture of hmvECs in cell crowns

For static culture, vascular grafts were placed in a cell crown culture system consisting of two metal rings according to Moll et al. [16] Therefore, sterilized vascular scaffolds were opened on one side, cut in pieces of approximately 1 cm^2^, and placed onto the smaller inner ring of a metal crown. Following, scaffolds were clamped by placing the bigger outer ring above the inner ring. Thereby, the vascular grafts were fixed in the middle of two rings, creating a so-called cell crown. The cell crowns containing the vascular grafts were then placed in 12-well plates. For seeding, hmvECs were detached using trypsin/EDTA solution. 4 x 10^5^ hmvEC per cell crown were seeded onto the vascular graft scaffolds. The cell crown was then filled with 0.75 mL Vasculife^®^ (containing 1% Pen/Strep) and the outer well was filled with 1 mL of the same medium. For further static culture, plates were placed in an incubator (37°C, 5% CO_2_, 95% rH). The culture medium was replenished every 2 days.

### Continuous flow perfusion bioreactor system

For dynamic cell culture tests, electrospun vascular grafts were cultured in a custom-made perfusion bioreactor system. The bioreactor harboring the graft was assembled by combining two 15 mL Falcon tubes and silicone tubes. All parts were washed and Gamma-sterilized before assembling the bioreactor. Tubular scaffolds were inserted into the bioreactor under sterile conditions and mounted to respective connectors using ligature suture. Following, a sterile silicone tube was used to seal the bioreactor. Thereby, an extra luminal compartment was generated. The bioreactor system enabled distinct extra and intra luminal flow conditions.

At the in- and out-let of the bioreactor, a three-way valves were attached to enable controlled filling of the graft with cell suspension and to facilitate intra and extra luminal perfusion. hmvECs were harvested and 2 x 10^6^ cells were transferred in 1 mL Vasculife^®^ into the lumen of 4-cm-long scaffolds. Moreover, the extra luminal compartment was filled with cell culture medium. To allow cell attachment, the seeded scaffolds were cultured under static conditions without flow in an incubator. Every 30 min, the whole bioreactor was turned for 180° to enable gradual attaching of the hmvEC onto all sides of inner lumen of the graft. After 4 hours, the intra and extra luminal compartment of the bioreactor were perfused with a continuous flow of 4.9 mL/min. Therefore, cell culture medium was pumped from a medium reservoir through the bioreactor by using an ISMATEC^®^ pump (MCP-Z Process, Glattbrugg, Switzerland). The medium reservoir was equipped with a sterile filter to allow gas exchange. The bioreactor was operated in a tailored incubator system [17] that ensured controlled culture conditions of 37°C and 5% CO_2_. During culture, 30 mL Vasculife^®^ medium was used and renewed once a week.

### Cell viability, functionality and morphology

Cell viability was tested on different vascular grafts both after static cell culture and cell culture in the continuous flow perfusion bioreactor system. To perform tests after dynamic cell culture, the vascular grafts were cut in pieces of 1 cm^2^ to enable a better comparison with samples after static cell culture. To test viability, functionality, and morphology a MTT assay (3-(4,5-dimethyl-2-thiazolyl)-2,5-diphenyl-2H-tetrazolium bromide), a Live/Dead assay, an AcLDL test (acetylated-low density lipoprotein), and immunofluorescence staining against collagen type I as well as a phalloidin staining of actin fibers were performed.

For MTT assay, all samples were transferred in 12-well-plates and incubated in MTT reagent (1 mg/mL in cell culture medium) for 90 min. To dissolve the formazan crystals, acid isopropanol (100 μL of 0.04 N HCL) was added. After a few minutes at room temperature, to ensure that all dark blue crystals were dissolved, 200 μL was added to 96-well plates and the plates were read with a Tecan infinite M200 reader, using a wavelength of 570 nm, a reference wavelength of 630 nm, and a calibration setting of 1.99 (or 1.00 if the samples were strongly colored).

The Live/Dead assay is suitable for determining live and dead cells after seeding and incubation on vascular grafts. Live/Dead solution consisted of fluoresceindiacetat (FDA), staining only the intracellular esterase activity of living cells, and propiumbromiodide (PI) (both Sigma Aldrich, Germany) staining only the plasma membrane disintegration of dead cells. The solution is prepared adding 1 μL of FDA (0.5 mg/mL) and 9 μL PI (0.05 mg/mL) into 990 μL PBS^-^. After allowing the Live/Dead solution to penetrate in the vascular grafts, the samples were washed with PBS^-^ and examined immediately under a fluorescence microscope (Leica, Wetzlar, Germany).

An AcLDL test was performed to metabolically label endothelial cells using acetylated-low density lipoprotein (acLDL), which is taken up by endothelial cells via the “scavenger cell pathway”. The acLDL, which is fluorescently labeled with Alexa Fluor 488 (by Life technologies), was added to the Vasculife^®^ medium to achieve a concentration of 10 μg/mL. Also 10 μL/mL of hoechst (by Life technologies, Carlsbad, California, USA) was added to cell medium to visualize the cell nuclei. After a 2 h incubation and a washing step in PBS^-^, tissue samples were analyzed under a confocal microscope (Leica, Wetzlar, Germany).

To determine the location of collagen fibers in vascular grafts, immunofluorescence staining was performed using collagen type I antibody (Acris Antibodies GmbH, Herford, Germany). Frozen section technique was employed using cryo-embedding media (Tissue-Tek, O.C.T., Sakura, The Netherlands). Samples were cut in a cryotome cryostat (-20°C) (Leica CM1860, Leica Biosystems, Germany).

Phalloidin staining of actin fibers inside the seeded endothelial cells was conducted by adding phalloidin (Molecular Probes—Life technologies, Eugene, Oregon, USA) dissolved 1:40 in PBS^-^ on samples fixed in 4% PFA. After 20 minutes of incubation, all samples were washed with PBS^-^ and after DAPI staining for cell nuclei, observed under confocal microscope (Leica CM1860, Leica Biosystems, Germany).

### The antibacterial efficacy of the vancomycin/gentamicin electrospun grafts in vitro

To investigate the possibility of improving the antibacterial efficacy of the grafts, the middle layer of the vascular grafts consisting of PCL was loaded with vancomycin/gentamycin. Therefore, 15% vancomycin (Hikma Farmacêutica, Portugal) and 6% gentamicinsulfat (Merck Serono GmbH, Darmstadt, Germany) were added to the PCL solution and the electrospinning process was performed as described. For antibacterial efficacy tests, *S*. *aureus* and *S*. *epidermidis* were chosen as the experimental bacterial strains. The *S*. *aureus* strain (DSM-799 from DSMZ–German Collection of Microorganisms and Cell Cultures) and *S*. *epidermidis* (O-47 from The Institute for Molecular Infection Biology, University Wuerzburg) were used to inoculate a Mueller-Hinton (MH) plate uniformly. Specimens were placed individually on each plate and consisted of two vascular grafts made of PCL/collagen/vancomycin/gentamycin and two PCL/Collagen type I vascular grafts as negative control. After incubation at 37°C for 24 h, measurements were taken to determine the diameter of the inhibition zone. This step was repeated for 4 weeks on a daily basis. To test cytotoxicity, grafts were placed in cell crowns and seeded with hmvECs to perform a MTT assay as described above.

### Haemolysis test and haemocompatibility test of PCL/collagen electrospun scaffolds

Peripheral blood samples were obtained under informed and written consent according to ethical approval granted by the institutional ethics committee of the Julius-Maximilians-University Wuerzburg from the Bavarian Red Cross blood donation service (Blutspendedienst des Bayerischen Roten Kreuzes, München, Germany). The study was approved by the ethics committee (vote 182/10). For the hemolysis tests, blood was diluted 1:150 with PBS^-^. Pieces of PCL/collagen scaffolds with and without mvECs, polyester and parafilm (polyolefin with paraffin) were incubated in 10 ml diluted whole blood in falcon tubes at room temperature (RT) under shaking conditions. Whole blood was diluted in distilled water or PBS^-^ without test material and served as positive and negative control, respectively. After 24 h, material was removed and the suspension centrifuged (200 x g, 5 min). Optical density of free haemoglobin in the supernatant was measured at 415 nm with a Tecan infinite M200 reader (Tecan, Crailsheim, Germany). Each experiment was carried out in triplicates (n = 3). Results were given as percentages, where deionized water showed maximal hemolysis (100%).

To evaluate coagulability of electrospun PCL/collagen graft material, cell-free scaffolds and scaffolds seeded with mvEC were prepared in a 96-well plate while polyester and polyolefin were considered as reference material. Blood was centrifuged at 2500 x g for 15 minutes and extracted plasma was diluted with 15 mM CaCl_2_ to reactivate coagulation. Plasma diluted in Thromborel S served as positive control, as negative control plasma was diluted in distilled water. Three replicate wells were used for each measurement. Optical density was measured at 320 nm with a Tecan infinite M200 reader (Tecan, Crailsheim, Germany) every 90 s for 40 min.

### Statistical analysis

Data were expressed as mean ± standard deviation (SD). Data was analyzed on normal distribution using the Kolmogorov-Smirnov-Test. Statistical analysis was performed using an unpaired student’s t-test and ANOVA analysis of variance. A p-value of p < 0.05 was considered as statistically significant.

## Results

### Generation of multilayered electrospun vascular scaffolds in a bidirectional electrospinning device with controlled process conditions

A multilayered porous nanofibrous vascular scaffold was manufactured in a custom-made device that allowed bidirectional gradient electrospinning (Fig 1A). To optimize the conditions during electrospinning and to identify the impact of the humidity on the scaffold properties, the spinning process was repeated at different humidity levels (30%, 50%, and 80%). Scanning electron microscopy (SEM) revealed that a low humidity (30%) results in the production of porous fiber structures (Fig 1B), whereas a higher humidity (50% and higher) leads to merged fibers without a detectable porosity (Fig 1C and 1D).

The tailored electrospinning system facilitated the fabrication of a multilayered scaffold with a diameter of 6 mm (Fig 1F). Therefore, pure PCL, pure collagen type I, or defined combinations of both materials were either spun separately or simultaneously (Table 1). To note, the scaffold diameter of 6 mm was given by the used collector mandrel and can be adjusted by adapting the mandrel diameter. Following electrospinning, collagen type I inside vascular graft was reinforced by intrafibrillar cross-linking of the collagen chains. Immunofluorescence staining of collagen type I in cross-sections of the scaffold wall demonstrated a symmetrical gradient structure, where the collagen layers could be detected at the intra and extra luminal surfaces (Fig 1F). The collagen gradient inside the scaffold wall was confirmed by image analysis of the fluorescence intensity, here exemplarily shown for the intra luminal surface (Fig 1G). Thereby, it was found that the presence of collagen was limited to a distance of approximately 40 μm from the intra luminal surface.

### Morphology, biocompatibility and biomechanical properties of multilayered porous nanofibrous electrospun vascular scaffolds

**Fig 2:**
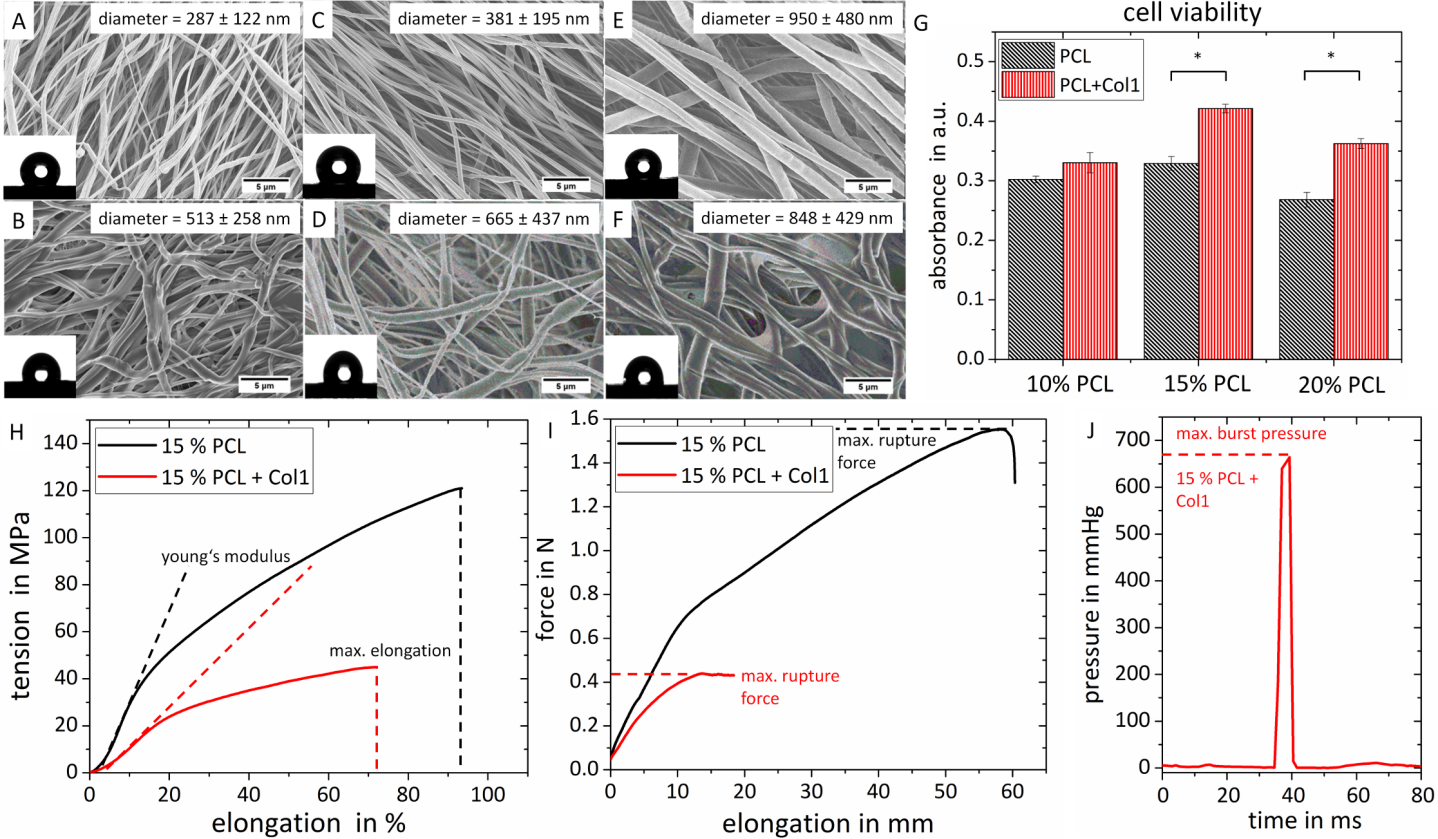
Scanning electron micrographs, biomechanical properties of vascular scaffolds and cell viability. (A-F) Microstructure and morphology of scaffolds luminal surface was visualized under SEM. The surface wettability of each scaffold is showed in corresponding inset. (A,C,E) PCL scaffolds (concentration 10%, 15%, and 20%). (B,D,F) Multilayered scaffolds with addition of collagen type I to inner and outer layer with the same PCL concentrations (10%, 15%, and 20%). Exemplarily, the luminal layer is shown. To calculate the fiber diameter, three individual images of the luminal surface were analyzed. (G) Cell viability was evaluated using a MTT assay that showed a higher cell viability on multilayered scaffold compared to PCL scaffolds (n = 3). (H) Tension-elongation curves, (I) suture elongation strenght, and (J) burst pressure tests for scaffolds electrospun with 15% PCL ± collagen type I. Data are expressed as mean ± standard deviation (SD), * indicates significant differences (p-value < 0.05).

To adapt the mechanical properties of the vascular scaffolds, the composition of the inner collagen-free scaffold component was tested at different poly-ɛ-caprolactone (PCL) concentrations. SEM images allowed the comparison of the microstructure and morphology of PCL scaffolds with 10%, 15% and 20% PCL (Fig 2A, 2C and 2E), and multilayered electrospun scaffolds with an inner and outer wall layer composed of collagen type I. For the latter the medial layer was manufactured with PCL at concentrations of 10%, 15% and 20% (Fig 2B, 2D and 2F).

The fabricated PCL and multilayered PCL/collagen vascular scaffolds exhibited fibrous structures (Fig 2A–2F). Moreover, the addition of collagen type I resulted in an increase of the fiber thickness. The evaluation of the mesh porosity of 3 individual samples of each scaffold revealed maximum pore sizes of 10.66 μm^2^ for 10% PCL, 51.10 μm^2^ for 15% PCL, and 54.34 μm^2^ for 20% PCL scaffolds. For the multilayered electrospun scaffolds, a robust measurement of the pore size was not applicable due to the formation of merged fiber structures. Nevertheless, crosslinking of collagen type I resulted in smaller pore sizes in the collagen/PCL scaffolds compared to PCL structures.

Since the hydrophobicity of a material has a significant impact on cell adhesion, proliferation and migration [18], the surface wettability of the generated scaffolds was measured (Inset in Fig 2A–2F, n = 6). Pure PCL vascular grafts exhibited higher hydrophobic properties than multilayered vascular grafts with an average water contact angle of 130.3° ± 2.1°, whereas hydrophilicity of collagen-blended vascular grafts increased significantly with an average contact angle of 102.1° ± 1.7°, as the surface of multilayered vascular grafts consisted of collagen type I.

The cytotoxicity of fabricated PCL and multilayered PCL/collagen vascular scaffolds was estimated by cell viability MTT analysis. The quantitative colorimetric assay revealed no statistical significant differences between electrospun vascular grafts made of PCL in different concentrations. However, there was a statistically higher rate of tetrazolium reduction (p < 0.05) by hmvECs seeded on multilayered vascular grafts containing PCL/collagen type I compared to those seeded on PCL vascular grafts (Fig 2G). This indicates a higher cell viability on multilayered PCL/collagen type I vascular grafts compared to PCL scaffolds. Following implantation, the grafts must endure physiological pressures and shear forces. To assess the mechanical properties, electrospun scaffolds were subjected to uniaxial elongation in order to evaluate tensile strength and elasticity (Fig 2H). Uniaxial tensile tests showed that the vascular grafts have an initial elastic behavior, followed by stiffening. This characteristic is similar to native arteries [19]. The PCL vascular scaffolds revealed a superior tensile strength and higher maximum elongation compared to multilayered PCL/collagen scaffolds. The suture retention strength of 15% PCL and multilayered vascular graft (15% PCL and collagen type I) was 1.62 ± 0.05 N and 0.43 ± 0.8 N, respectively (Fig 2I). Suture retention strength for PCL scaffolds were comparable to results previously reported and comparable to the suture retention strength of a saphenous vein. Multilayered vascular scaffolds showed lower suture retention compared to the results of native arteries reported by other investigators. [20] Burst pressure test showed that the maximal burst pressure strength for multilayered vascular grafts consisting of 15% PCL and collagen type I was 664 mmHg (Fig 2J).

**Fig 3:**
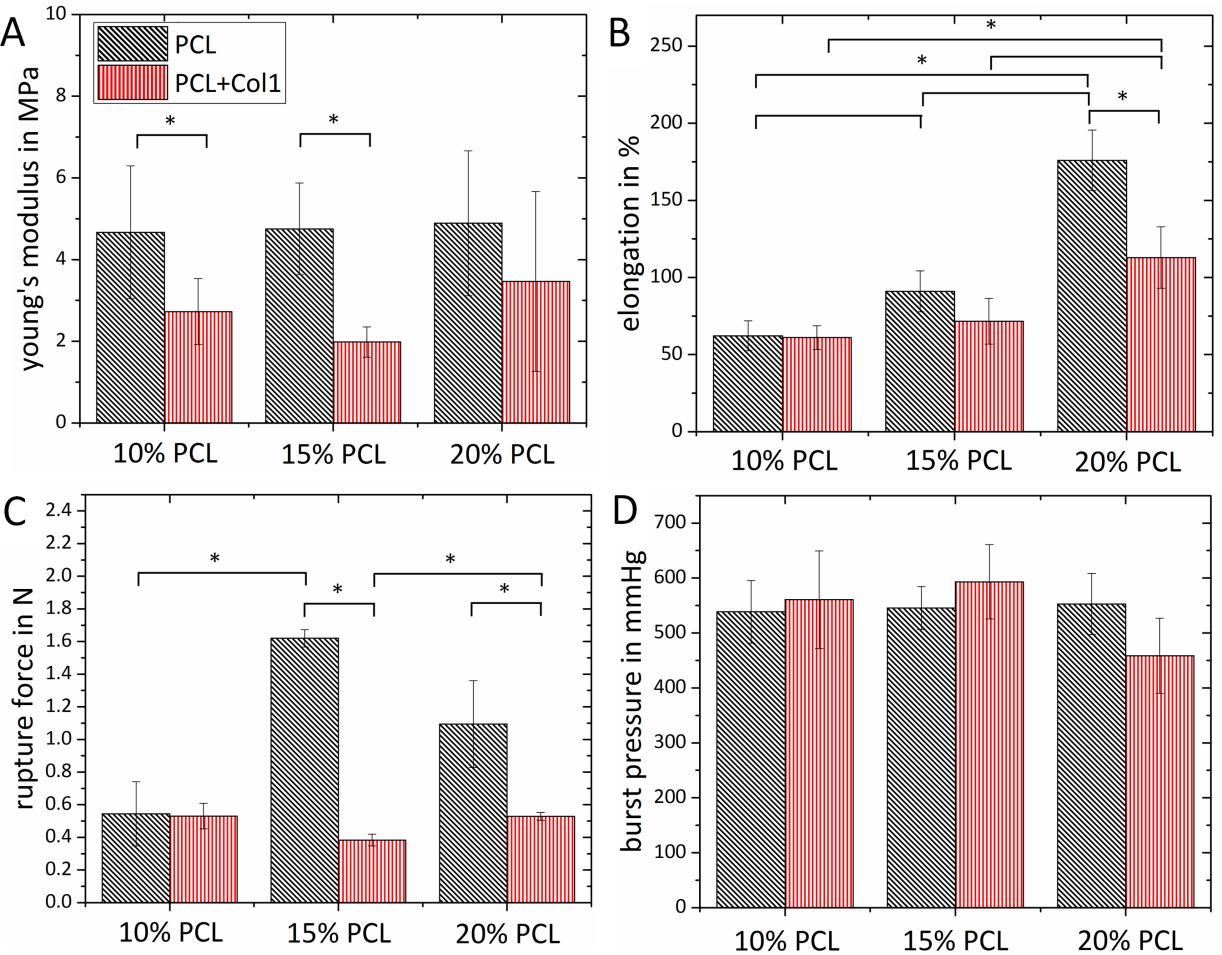
Comparison of the mechanical properties of electrospun scaffolds produced from different PCL concentrations. (A) Young’s modulus (n = 3), (B) maximum elongation (n = 3), (C) suture retention strength (n = 3), and (D) burst pressure (n = 4). Data are expressed as mean ± standard deviation (SD), * indicates significant differences (p-value < 0.05).

When comparing the mechanical properties of the different scaffolds, no impact of the PCL concentration on the young’s modulus was found (Fig 3A). Compared to the PCL scaffolds, the multilayered scaffolds showed a decreased young’s modulus. For 10% and 15% PCL, the reduction was statistically significant. In contrast to the young’s modulus, the maximum elongation was significantly increased with increasing PCL concentration (Fig 3B). This effect was weakened for the multilayered grafts. In analogy to the maximum elongation, also the suture retention strength of depended on the PCL concentration. Hereby, the highest suture retention strength of PCL scaffolds was found for a concentration 15% PCL. Alike the young’s modulus and the maximum elongation, a multilayered scaffold architecture also impaired the suture retention strength. For the burst pressure, neither a dependency on the PCL concentration nor on the scaffold structure was found (Fig 3D).

Due to higher cell viability and hydrophilicity, multilayered PCL/collagen type I vascular grafts were used for further graft optimization.

### Cell seeding efficiency, viability and phenotypic expression under static and dynamic culture conditions

**Fig 4:**
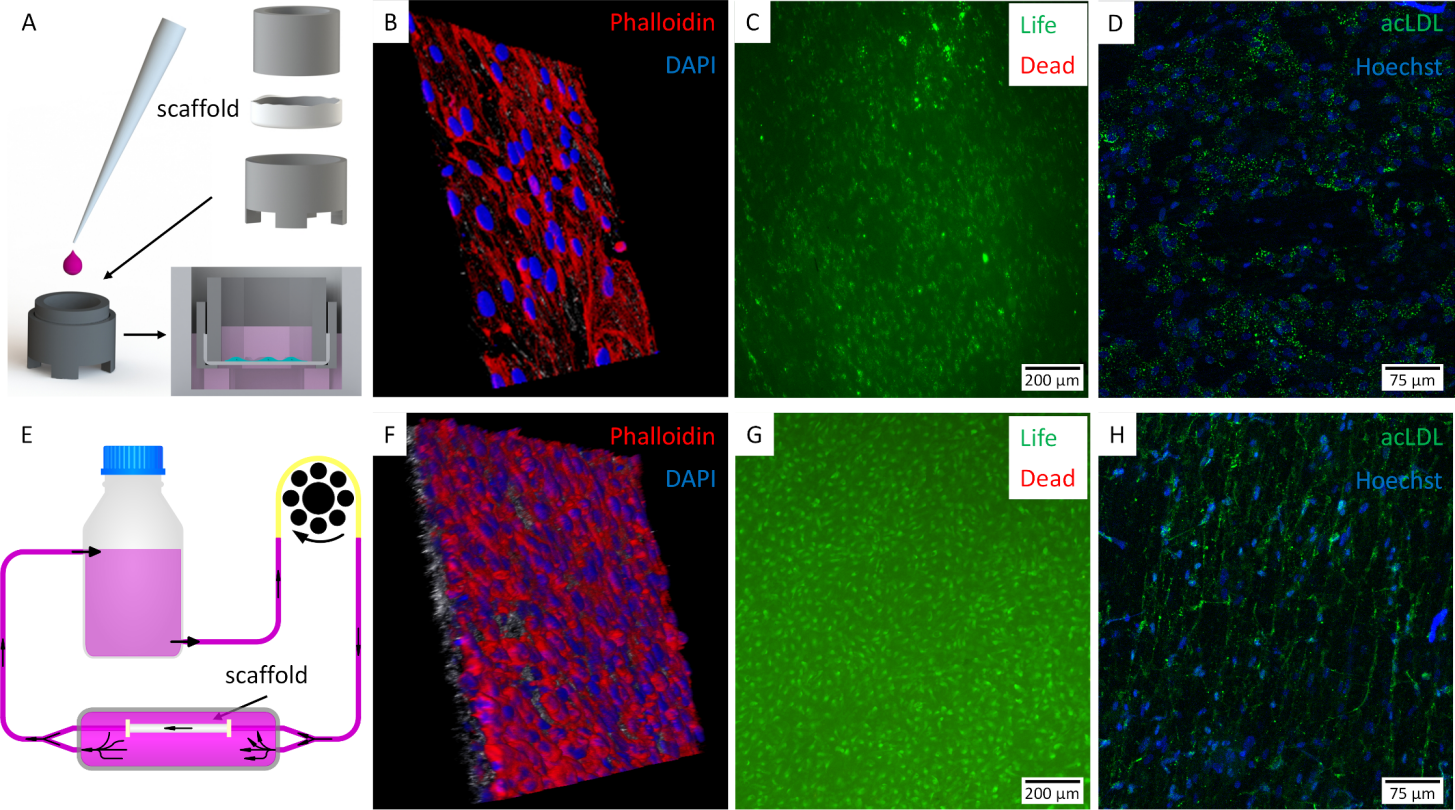
Cell seeding efficiency and viability under static and dynamic conditions. (A) Static cell culturing was performed in a cell crown culture system. A cell crown consisted of two tailored metal rings and facilitated the culture of cells on a membrane that is mounted between the two rings. A cell crown can be used comparably to a standard trans-well insert and can be placed in a 12- or 6-well plate. (B) Phalloidin staining detected actin filaments. (C) Seeded hmvECs remained viable after 7 days static culture. (D) Metabolic activity was demonstrated by uptake of acetylated LDL. (E-H) Dynamic culture of tubular vascular grafts was performed in a (E) perfusion bioreactor system inside a closed chamber with controlled environment conditions. (F) After 10 days of culture, phalloidin staining revealed a dense hmvECs layer of the whole inner circumference, with (G) vital and (H) metabolically active cells. The microscopic images in (B) and (F) depict a tissue segment of 300 x 300 μm.

For static culture, multilayered PCL/collagen type I vascular grafts were placed in a cell crown culture system [16] consisting of two metal rings and seeded with 4 x 10^5^ hmvEC (Fig 4A). After 7 days of culture, cell seeding efficiency and viability was assessed. Using phalloidin staining, actin filaments of seeded cells were detected (Fig 4B). The staining showed that hmvECs organized in a monolayer on the surface of the vascular grafts and Live/Dead staining confirmed cell viability (Fig 4C). Furthermore, acetylated-low density lipoprotein (Ac-LDL) was used to label hmvEC and to prove metabolic activity; especially the clearing function of the scavenger pathway (Fig 4D).

In addition to the static culture in the cell crowns, tubular vascular grafts were exposed to dynamic conditions. Therefore, scaffolds were filled with cell suspension containing 2 x 10^6^ hmvECs and incubated in the perfusion bioreactor system (Fig 4E). After 10 days of culture, a closed cell layer around the whole circumference was achieved (Fig 4F). More than 98% of these cells were vital (Fig 4G). Phenotypic uptake of acLDL via the scavenger cell pathway (Fig 4H) confirmed cell identity and an endothelial-specific metabolic activity on the inner layer of the graft. Furthermore, when static and dynamic cultures were compared, an improved homogeneity of hmvECs was achieved in the dynamically cultured grafts (e.g. Fig 4B vs Fig 4F), whereby cells were organized in flow direction (Fig 4H). These results indicated that the dynamic flow conditions stimulated an increased cellular adhesion and organization on the luminal side of the grafts while maintaining hmvECs phenotype and viability.

### Bioactivity of antibiotics-loaded vascular grafts

**Fig 5:**
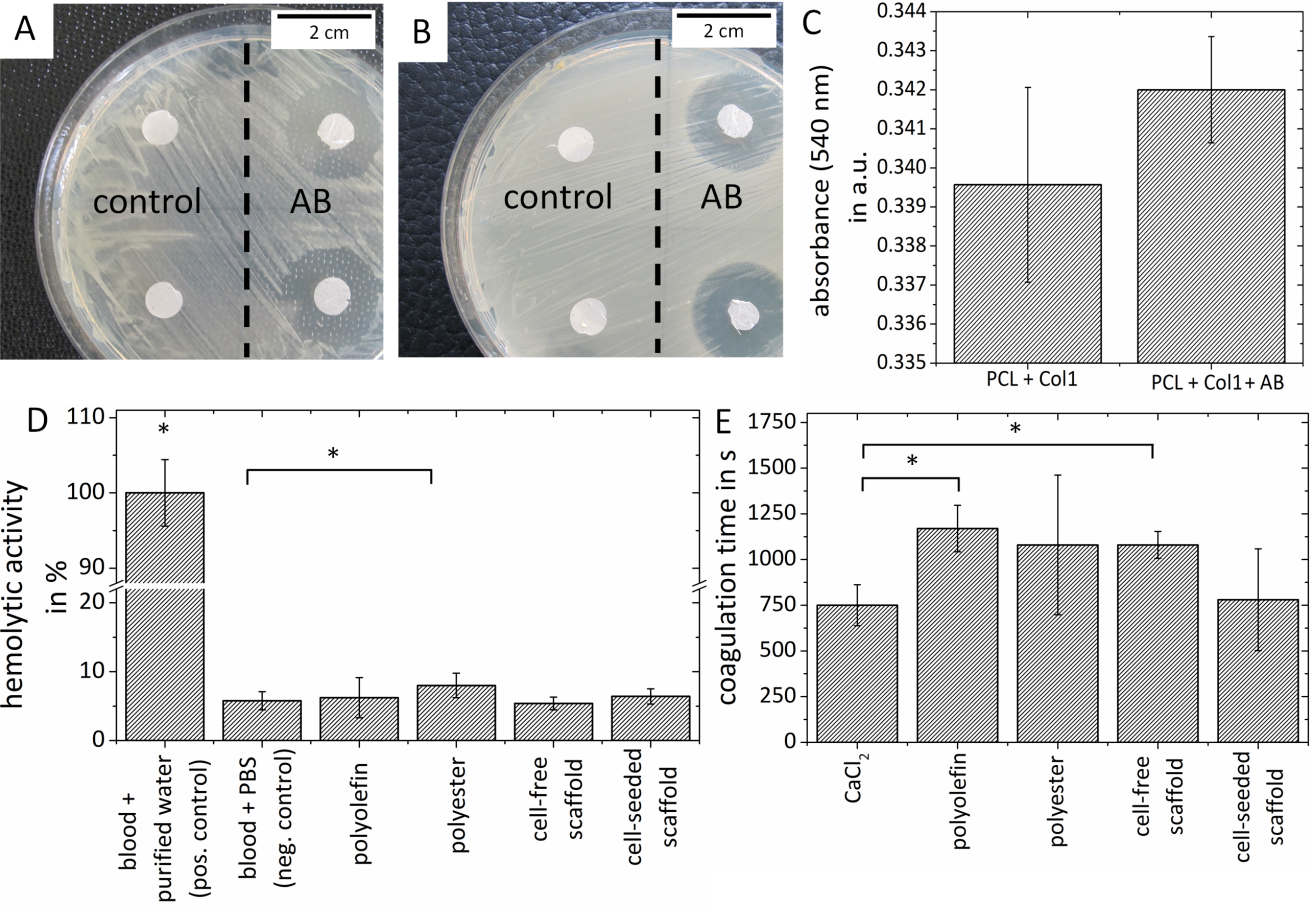
Bioactivity of antibiotics loaded vascular grafts and hemocompatibility test. Antimicrobial resistance of antibiotics-loaded vascular grafts on Muller Hinton agar plates, against (A) *S*. *aureus* and (B) *S*. *epidermidis*. (C) Quantitative MTT assay comparing hmvECs viability on vascular grafts loaded with vancomycin and gentamicin compared to multilayered grafts (n = 3). Hemocompatibility of multilayered grafts was tested using a (D) hemolysis test and a (E) coagulation test. Data are expressed as mean ± standard deviation (SD), n ≥ 3, * indicates significant differences (p-value < 0.05).

Electrospun vascular grafts can be combined with antibacterial agents to reduce the risk of vascular graft infection. The effect of grafts loaded with vancomycin and gentamicin was evaluated in bacteria inhibition experiments. Initially, the maximum mean diameter of the inhibition zone in the presence of *S*. *aureus* and *S*. *epidermidis* was 14.5 ± 0.48 mm and 20.5 ± 0.5 mm (n = 3) on the first day, respectively (Fig 5A, Fig 5B). Over the next 28 days, the diameter of the inhibition zone increased and remained unchanged over the observed period of 28 days.

Furthermore, it was confirmed that the addition of antibiotics had no negative effect on hmvEC adhesion and proliferation. In a qualitative MTT assay, vascular grafts loaded with vancomycin and gentamicin and seeded with hmvECs facilitated cell viabilities resulting in a comparable rate of tetrazolium reduction like vascular grafts without antibacterial loading. No statistical significant difference was detected between both grafts. (Fig 5C).

### Hemocompatibility of tissue-engineered vascular grafts

To assess the hemocompatibility of the generated vascular grafts, a blood lysis test and a coagulation test were performed. The hemolysis test results are represented as relative values to the positive control (blood sample in purified water), which induced a 100% hemolysis (Fig 5D). While the human whole blood incubated with polyester revealed higher levels of soluble hemoglobin compared to blood diluted in PBS^-^ (negative control), the hemolytic activity of PCL/collagen type I vascular grafts (seeded and cell-free) after contact with diluted blood was low and comparable to the negative control (Fig 5D).

In the coagulation test, CaCl_2_ was diluted with citrate-buffered human blood extracted plasma to reactivate coagulation. This experimental condition served as physiological coagulation control. The positive control—Thromborel S—was already clotted before reading the results in the absorbance reader and a negative control–blood diluted in distilled water—had not shown any clotting during the observed period over 30 min. Thus, data for Thromborel S and distilled water are not provided in the results. Polyester and polyolefin are described to provide a good blood compatibility, and were chosen as reference material. [21] No difference was detected between the physiological coagulation and PCL/collagen vascular grafts seeded with hmvECs (Fig 5E). Cell-free vascular scaffolds showed a coagulation characteristics similar to polyesters, material already used as vascular grafts or suture material, and a slower coagulation time compared to the physiological time.

## Discussion

The population of patients with end stage kidney disease is growing and suitable vascular access remains a major medical problem in medical intervention. [22] An arteriovenous fistula represents first choice for hemodialysis access. When this option is not available, prosthetic grafts such as polytetrafluoroethylene (ePTFE) serve as alternative. However, prosthetic grafts are associated with an increased morbidity and mortality [23] and they don’t match the properties of autologous blood vessels, particularly for diameters ≤ 6 mm. [24, 25] Tissue engineering provides novel options for the generation of vascular grafts with more or less success in the past decade. [19, 26] Production of suitable dialysis grafts mimicking native vessel structure beside of being a challenge itself, needs to address further requirements such as: suitable handling during implantation, non-thrombogenic properties, biocompatibility, resist aneurysmal dilatation, and the ability to lower the risk of onsite infection after implantation despite repeated needle trauma. The work presented here, demonstrates that vascular endothelial cells can be seeded on electrospun PCL/collagen type I vascular grafts and proliferate in a dynamic bioreactor with continuous flow. The graft maintained the structural integrity during the period of incubation without any malformation, rupture or aneurysmal change. Moreover, we were able to combine these vascular grafts with typical antibiotics and prove resistance against *S*. *aureus* and *S*. *epidermidis* as typical pathogens for onsite infection. Most striking a physiological hemocompatibility and suitable mechanical properties were demonstrated for the multilayered grafts.

Vascular grafts were generated using a bidirectional electrospinning with controlled process conditions. Electrospinning is an effective and cost-efficient method, which uses electrical force to draw charged threads of polymer solutions to produce micro- or nanofibers with suitable porosity that enables a good cell adhesion and growth. For this process, we developed a custom-made device allowing bidirectional gradient electrospinning of two different polymers from opposite spinnerets on a rotating mandrel. The production of multilayered vascular grafts was previously described by Xiumei Mo and his group, who used different polymer solutions in separate syringe pumps placed opposite to each other to perform bidirectional electrospinning. [27] Due to the bidirectional electrospinning, each layer can be tailored to achieve specific characteristics such as mechanical strength or specific wettability. In this study, sequential electrospinning was performed, whereby the collagen type I and PCL flow ratio increased and decreased simultaneously, so that at the beginning and the end of the process only collagen type I and in the middle only PCL was deposited. As a result, the luminal and outer layer were made only of natural material–collagen type I. Collagen type I was favored as the major component of a native vessel and due to it’s described biocompatible properties. PCL, a medically-approved elastic polymer, allowed to tailor graft strength in order to endure physiological hemodynamic conditions. [19] The mechanical characteristics of native arteries are associated to the collagen and elastin content as well as the presence of smooth muscle cells, which are all parts of an arterial wall. Collagen in blood vessels provides mechanical strength and prevents rupture, elastin provides recoil after each inflation and prevent dilatation of arteries and together with smooth muscle cells regulates arterial compliance. [28] The first collagen based vascular grafts obtained by electrospinning were produced by Huang et al. who used collagen in combination with Polyethylene Oxide. [29] Pure collagen layers in the graft were not fabricated. Additionally, it was shown that SMC and ECs prefer collagen over synthetic materials. However, given a high intraluminal pressure, it is likely that pure collagen vascular grafts lead to mechanical failure. [30, 31] Combination of PCL and collagen electrospinning has already been tested, but most of this work is based on either coating the collagen layer on electrospun PCL, where the risk of delamination is higher, or mixing the both polymers in one solution and electrospinning them together on collector. [19] Also electrospinning of natural proteins has been described when Buttafoco with his group electrospun collagen together with elastin, however mechanical test results were not reported. [32]

Since relative humidity plays a key role on ultrafine morphology of the vascular grafts fibers in electrospinning [33], in our device the whole process was performed in a closed chamber with the controlled process conditions. Analyzing SEM images, it was found that the most suitable porous structure is produced, when the relative humidity stays below 30%. The relative humidity during vascular graft production was controlled to be 27 ± 2% and temperature 22°C. Through the SEM, we confirmed that both PCL and multilayered PCL/collagen type I vascular grafts, electrospun under these conditions, were formed of continuous nanofibers under all solution concentrations and flows used in the study. Fibers in the multilayered vascular grafts revealed a higher diameter without changes in pore sizes, in contrast to PCL vascular grafts, thereby representing a suitable substrate for cell attachment and growth of hmvECs. Furthermore, the addition of a collagen layer onto a PCL structure in the multilayered grafts significantly increased hydrophilicity, which had a positive effect on cell adhesion, proliferation and migration. Changing the temperature and humidity conditions in the electrospinning chamber resulted in changes of vascular graft microstructure. Thicker fibers with smaller pores were produced on the luminal side and bigger pores on the outer layer allowing proliferation and migration in the vascular graft.

The ANSI guidelines (Association for the Advancement of Medical Instrumentation American National Standards Institute. Cardiovascular implants—Tubular vascular prostheses. Arlington, VA: AAMI; 2004.) recommend measuring longitudinal and circumferential force of the vessels, burst pressure, and suture retention strength. PCL vascular grafts showed superior tensile strength compared to multilayered grafts and higher elongation properties, which should prevent graft inflation when pressure is increased. The results for the PCL scaffolds were comparable to results reported by other authors. [34] Multilayered vascular grafts showed tensile strength inferior to PCL, with lower elongation properties. Similar results were reported from groups who blended these two polymers and showed higher elongation and tensile strength in PCL scaffolds compared to ones blended with collagen type I. [35] Young’s Moduli were calculated for the multilayered grafts by assuming a material thickness of 10 μm. PCL scaffolds showed a higher elastic modulus compared to multilayered vascular grafts as shown in Fig 3A. Similar results were demonstrated by Xiumei Mo and his group, when comparing pure PCL and multilayered scaffolds with an additional outer layer composed of chitosan. They also showed different mechanical properties of grafts in dry and wet state, demonstrating results favoring multilayered scaffolds in wet state. [27] All our mechanical tests were performed in dry state.

Further testing of burst pressure and suture retention strength are important to ensure that the graft can withstand the surgical manipulation during implantation and physiological pressure after implantation. The average pressure in the arterial circulation is 100 mmHg, and is higher in leg arteries while standing. Due to the contribution of hydrostatic pressure, pressure values can reach up to 250 mmHg. [36] In our experiments, despite different tensile strength properties, we have shown no significant difference in pressures, which led to graft failure between PCL and multilayered grafts. Mrowczynski et al. showed good surgical friendliness and in vivo characteristics of pure PCL grafts when implanted in rat’s infrarenal artery and porcine carotid artery without graft failure due to physiological pressure. [37] The mechanical stiffness and burst pressure needed for arterial implantation is not defined but studies have shown that burst pressures of 600–700 mmHg can be implanted without significant dilatation in porcine models. [38] In our study, multilayered vascular graft containing 15% PCL and collagen type I sustained max. 664 mmHg (Fig 2J). This multilayered scaffold was used further on, for in vitro dynamic seeding with hmvECs. When considering suture retention strength, multilayered grafts showed a lower force required to pull the suture through the graft compared to pure PCL sutures, which were comparable to the values of native vessels ranging between 0.8 N to 2.5 N reported in other studies. [39] In further experiments, we will consider increasing the thickness of the middle PCL layer, which might lead to even better suture retention strength in multilayered vascular grafts.

Beside the need of sufficient mechanical properties, the vascular graft needs to be biocompatible to accommodate vascular cells while maintaining structural integrity. In our study, we first used a cell crown systems to test adherence tendency of hmvECs towards different electrospun vascular grafts in static conditions. On both, PCL and multilayered vascular grafts, hmvECs adhered and proliferated to a closed cell layer. The statistically higher viability rate in multilayered vascular grafts was probably induced by a higher hydrophilicity and slightly smaller pore sizes, providing a higher surface area for cell adhesion. Due to this, we decided to use only multilayered vascular grafts for the dynamic bioreactor experiments. These scaffolds provided sufficient mechanical properties and superior cellular environment for grafting applications and to test in vitro capability of such vascular grafts to maintain cell adherence.

A rapid endothelial coverage of the surface, which was achieved in the seeding experiments, is mandatory as this surface is in direct contact with blood flow and is also necessary to offer anti-thrombosis property to acquire long-term patency as endothelial integrity represents important part of Virchow’s triad. [40] In the graft, endothelial cells completely covered the graft surface within one week of static culture, as well as after 10 days of culture under medium flow conditions. Hereby, it is important to stress that the incubation time in the dynamic bioreactor was 10 days compared to 7 days static cell culture. Although a longer culture duration was performed in contrast to static culture, the required dynamic culture period is shorter compared to durations reported by other groups, who performed 14 days incubation preceded with 3 days static cell culture. [16, 17] In our study, we demonstrated a sufficient intimal layer coverage after 10 days dynamic cell culture in a flow reactor, which was preceded with 4 hour static cell culture. Phenotypical and functional assays on vascular grafts seeded with endothelial cells were performed to assess the properties and function of the blood-tissue interface. After incubation in dynamic flow bioreactor vWF and CD31 expression was detected, as one of characteristics to confirm endothelial identity. Staining the actin fibers of endothelial cells using phalloidin showed tendency of endothelial cells to organize in one single layer and to fully cover the luminal side of the graft. Uptake of acetylated LDL through scavenger receptor mediated endocytosis was tested and confirmed. This proved that endothelial cells in the electrospun vascular grafts remained functional and were able for active transport of molecules. [41, 42] Moreover, it was detected that endothelial cells were organized and aligned in flow direction. Such organized continuous layer of endothelial cells represents an important atheroprotective factor. [43]

An important aspect of this study is vascular graft infection. The incidence of graft infection is reported between 1% and 6% [44, 45], whereas mortality varies between 25% to 75% and plays one of the most important reason for death of dialysis patients. [46, 47] Hence, the combination of electrospun vascular grafts with antibacterial agents to reduce the risk of infection was investigated. Different approaches based on repelling or directly killing pathogenic agents were investigated regarding their capacity to reduce implant related infections. Silver ions were successfully coated with polymeric materials to cover the surface of vascular grafts prior to implantation. [48] Although colonies of *S*. *aureus* were successfully inhibited without signs of cytotoxicity, the concept of covering the surface of vascular grafts with silver ions might compromise the possibility of seeding smooth muscle cells on top of these grafts in further studies. Moreover, in cases of diverse surgical site infections, different antibiotics were assessed regarding their ability to exhibit a local antimicrobial effect. [49–51] In our study, antibiotics were added to the PCL layer between the two collagen-blended layers as the collagen type I surfaces exhibited a suitable characteristics for endothelial growth. The bacterial growth inhibition experiments demonstrated that vancomycin and gentamicin can be added in a PCL solution and that their antimicrobial activity is unaffected by the electrospinning process. Such vascular grafts loaded with antibiotics are capable to inhibit growth of *S*. *epidermidis* and *S*. *aureus* in vitro, which are known as most common bacteria in surgical graft infections. [52–54] Using the MTT test, it was shown that vascular grafts coated with antibiotics facilitated cell viabilities comparable to vascular grafts without antibiotics. The detected higher viability for the graft blended with vancomycin and gentamicin was not statistically significant. This implicated that such grafts beside bacterial inhibition are not expected to have negative effect on host cell adhesion and proliferation. However, further tests are needed, particularly to test antibiotic release rate from such grafts in vitro and its efficacy in vivo after graft implantation. Hemocompatibility is the major criteria, when considering a biomaterial for cardiovascular applications. Hemocompatibility depends on the biomaterial’s surface and characteristics such as hydrophilicity and surface charge. These properties have a strong effect on the blood after an initial contact with the material. Thus, a successful vascular graft must be produced of a material, which does not cause any hemolysis and should not lead to hypercoagulability at the implantation site to prevent graft thrombosis. In addition to comparing multilayered vascular grafts to positive (physiological coagulation) and negative (inhibited coagulation) controls, the electrospun biomaterials were compared to polyolefin and polyester—two materials that are known for their hemocompatibility. Polyolefins are utilized in tubing’s and housings for blood supply such as blood bags or in heart valve structures. [21] Polyesters are also known under the name Dacron, and are often used as vascular grafts in modern surgery. We detected that neither the multilayered vascular scaffolds nor the vascular scaffolds seeded with hmvECs caused hemolysis, which would result in differences compared to polyolefin and polyester. Another important factor is blood coagulability after contact with vascular scaffold. We have seen that the cell-free multilayered vascular grafts showed similar results as the established vascular prosthesis material (polyester). Multilayered vascular grafts that were covered with hmvECs showed results comparable to CaCl_2_ activated plasma, which represented the physiological coagulation dynamics. Therefore, multilayered vascular grafts proved two fundamental hemocompatibility characteristics: they do not cause hemolysis after getting in contact with blood, and even cell-free scaffolds show lower coagulability allowing cell growth and endothelization without danger of graft thrombosis.

## Conclusions

This study shows that controlled electrospinning processes facilitate the production of vascular scaffolds for tissue engineering. The scaffold characteristics can be tailored by controlling the process conditions such as temperature or humidity. Multilayered PCL/collagen electrospun vascular scaffolds represent a suitable combination of synthetic and natural polymers, which can be seeded with hmvECs during incubation in dynamic continuous flow bioreactor. Endothelial lining inside vascular grafts was achieved and a hemocompatible blood-tissue-interface was obtained. Furthermore, the grafts provided a high degree of patency and structural integrity during in vitro incubation. When incorporating antibiotics into the graft scaffold, the susceptibility for post-operative infection is decreased. In summary, electrospun vascular grafts might have potential for clinical applicability as an alternative to traditional prosthetic graft materials, thereby overcoming problems of patients on hemodialysis who are lacking a suitable vascular access.
